# Women’s lived experiences of induction of labour in late- and post-term pregnancy within the Swedish post-term induction study – a phenomenological study

**DOI:** 10.1080/17482631.2022.2056958

**Published:** 2022-04-10

**Authors:** Helena Nilvér, Ingela Lundgren, Helen Elden, Anna Dencker

**Affiliations:** a Institute of Health and Care Sciences, Sahlgrenska Academy, University of Gothenburg, Gothenburg, Sweden; b Department of Health and Care Sciences, The Arctic University of Norway, Tromsö, Norway; c Region Västra Götaland, Department of Obstetrics, Sahlgrenska University Hospital, Gothenburg, Sweden

**Keywords:** Induction of labour, late-term pregnancy, post-term pregnancy, phenomenology, SWEPIS

## Abstract

**Purpose:**

There is a trend worldwide to induce pregnant women earlier. However, few studies have focused on women’s experiences. The aim was to gain a deeper understanding of women’s lived experiences of induction of labour in late- and post-term pregnancy.

**Methods:**

Phenomenology with a reflective lifeworld approach was chosen as the method. Twelve women participating in a larger study in which women were randomized to either induction of labour in week 41 or to expectant management until week 42, were interviewed one to three months after giving birth.

**Results:**

The essence is described as follows: *labour becomes another journey* than the intended one. The women adapted to this new journey by seeing the advantages and handing themselves over to the healthcare system, but at the same time something about giving birth could be lost. The result is further described by its four constituents: *planning the unplannable, being a guest at the labour ward, someone else controlling the labour*, and *overshadowed by how it turned out*.

**Conclusion:**

Induced labour presents a challenge to maternity personnel to support the birthing woman’s normal progress, not to rush her through labour, and to involve her in the process.

## Introduction

Childbirth has been described as an unavoidable situation for pregnant women (Lundgren, 2005) and tends to leave long-lasting impressions that follow them throughout life, as women often remember their childbirth experiences very well (Bossano et al., 2017; Simkin, 1991). Most women hope for a normal birth in a safe environment, with support from kind, sensitive, clinically competent staff (Downe et al., 2018). In previous qualitative research, it has been expressed how women in late-term pregnancy (pregnancy at or beyond 41 gestational weeks) may doubt their bodies’ capabilities to initiate labour and have increasing worries about their unborn baby (Wessberg et al., 2019). Late- and post-term pregnancy (pregnancy at or beyond 42 gestational weeks) is associated with a small, but increased, risk of perinatal death (Alkmark et al., 2020; Middleton et al., 2020). Therefore, there is an increasing trend to recommend induction of labour for healthy women in late- and/or post-term pregnancy.

Induction of labour is a common intervention in childbirth, aimed at starting the labour before spontaneous onset. Induction of labour tends to be presented to women in a way that compliance with guidelines and hospital routines is assumed and women’s trust in the opinion of health professionals appears to be strong (Jay et al., 2018). Previous studies have associated induction of labour with a more negative childbirth experience (Falk et al., 2019). Women who have their labour induced tend to have more worries and be less satisfied with their care compared to women with spontaneous onset of labour (Henderson & Redshaw, 2013). However, this has not been seen in research on women’s childbirth experiences in late- and post-term pregnancy (Hildingsson et al., 2011).

A late- or post-term pregnancy can be perceived by the individual woman as demanding. Therefore, induction of labour can be welcomed as a relief from the discomfort of the post-term pregnancy and a way to gain control over the unpredictable situation of pregnancy (Gatward et al., 2010; Moore et al., 2014). In a questionnaire study by Keulen et al. (2020), women who preferred induction in gestational week 41 (270/604) reported higher levels of anxiety and lower levels of quality of life than women who preferred expectant management until 42 gestational weeks (245/604). Reasons for wanting an induction included the fact that it provided a safe feeling and the experience that the pregnancy was taking too long. Meanwhile, for women who preferred expectant management, giving birth as naturally as possible was their main reason for not wanting an induction of labour (Keulen et al., 2020).

This study is part of the SWEdish Post-term Induction Study (SWEPIS; Elden et al., 2016; Wennerholm et al., 2019), in which women were randomized to either induction of labour at gestational week 41 (intervention group) or expectant management until 42 gestational weeks (control group). We have conducted one previous study on women’s childbirth experience in SWEPIS. Women in both groups reported similar childbirth experiences, measured with the Childbirth Experience Questionnaire version 2 (CEQ2) (n = 656) three months after birth, or overall childbirth experience assessed on a Visual Analogue Scale (VAS) (1-10) (n = 1457) within three days after birth (Nilvér et al., 2021).

There are a few qualitative studies on women’s experiences and perceptions of induction of labour from different perspectives in post-term pregnancy (Lou et al., 2018). However, we have only found one qualitative study focusing solely on women’s experiences of induction of labour in late- and/or post-term pregnancy. It is a newly published article from Denmark in which Lou et al. (2020), using thematic analysis, interviewed women with uncomplicated pregnancies about their experiences of late-term induction of labour. The results showed that the women had hoped for spontaneous onset of labour and that women reporting a positive childbirth experience emphasized good communication, feeling safe, and being cared for.

As there is an increasing trend to induce in late- and post-term pregnancy, it is important to know how women can perceive this experience. Therefore, the aim of this study was to gain a deeper understanding of women’s lived experiences of induction of labour in late- and post-term pregnancy.

## Methods

Phenomenology with a reflective lifeworld approach, as described by Dahlberg et al. (2008), was chosen as the method for this study as it is suitable for studying a phenomenon such as the experience of induction of labour. Lifeworld phenomenology aims to describe, clarify, and immerse our understanding of human experience in this world. Merleau-Ponty (2013) describes the lifeworld as “being in the world” and as “return to the things themselves”. The lived body is essential for the lifeworld, as it is through the body that we experience the surrounding world and it becomes meaningful for us.

Within phenomenology as a research approach, it is important to have an open attitude—a willingness to listen, see, and understand the phenomenon studied. To be able to do this, there is a need to reflect on and bridle one’s pre-understanding so that it is reflected upon and recognizes one’s own contribution to the research. This is achieved by not being hasty in understanding the phenomenon of interest but instead being open, respectful, sensitive, and alert by systematically and carefully exploring it (Dahlberg et al., 2008).

### Recruitment and procedure

Women participating in this study form a subgroup in the randomized SWEPIS (n = 2760; Wennerholm et al., 2019). Being a healthy woman at 41 gestational weeks, aged 18 or older, with a singleton pregnancy with a foetus in cephalic presentation was the inclusion criterion in SWEPIS. Women with previous caesarean section or uterine surgery, or known complications with their current pregnancy, were excluded. Women were randomized to either induction of labour at 41 gestational weeks (intervention group) or expectant management with induction at 42 gestational weeks if still pregnant (control group).

For inclusion in this study, we looked for variation in women’s experiences. We considered age, parity, any complications during birth, and induction in both gestational weeks 41 and 42. Women were sent an email with information about the study, and if they were interested in participating they were asked to respond to the email and were then contacted by phone for further information. The time and place for the interview were chosen by the women at their convenience. Before the interviews began, the participants were informed that participation was voluntary and that they had the right to withdraw their consent at any time without affecting their care, and that data would be handled confidentially. Before the interview began, the participants gave their written consent.

### Participants

Twelve women aged 29 to 42 years were interviewed between January and December 2018 about their experiences of labour induction. Eleven of the women were born in Sweden and one was born outside Europe. Eight women had their labour induced in gestational week 41, and four in gestational week 42. For seven of them this was their first labour, while five were multiparous. Nine of the women had spontaneous vaginal births, one had an instrumental vaginal birth, and two had an emergency caesarean section (see, Table I). Methods for inducing labour included cervical ripening with prostaglandins and/or Foley catheter, amniotomy, and infusion with synthetic oxytocin.

**Table I.** Labour outcome for participating women

	Spontaneous	Instrumental	Caesarean	Total:
Induction gestational week 41 Primiparous	3	1	1	5
Multiparous	3	0	0	3
Induction gestational week 42 Primiparous	2	0	0	2
Multiparous	1	0	1	02
Total:	9	1	2	12

Spontaneous—Spontaneous vaginal birth Instrumental—Instrumental vaginal birth Caesarean—Emergency caesarean section

### Data collection

Eight of the women chose to be interviewed at home and four in a secluded room at Sahlgrenska University Hospital. They were interviewed by HN between one to three months after giving birth. The interview started with the opening question “Can you tell me about your experience of having your labour induced?”, and follow-up questions such as “Can you tell me more?” and “How do you mean?” were asked in order to gain a deeper understanding of the woman’s experience. Clarifying questions were asked when necessary. The interviews lasted 26 to 110 minutes and were recorded and then transcribed verbatim. In the interview transcriptions, the women were given pseudonyms. All interviews were conducted and transcribed by the first author.

### Data analysis

The data was analysed using reflective lifeworld research as described by Dahlberg et al. (2008), in which the analysis aims to find structures of meaning that describe the phenomenon of interest.

The first step in the analysis was to become familiar with the text, reading and re-reading each interview to familiarize ourselves with the material and get a sense of it as a whole. After this the reading changed, with different parts beginning to emerge from the text and meaning units being identified. With an awareness of the text as a whole, we then organized the meaning units to see and understand patterns and clusters of meaning. This process initiated a movement back and forth in the material, reflecting on and transforming the clustered meaning units forming a pattern describing the phenomenon. Finally, the text was again treated as a whole, but now with a broader understanding, combining the meaning units into a new structure of meanings—the essence of the phenomenon. The meaning units were then further synthesized into constituents clarifying and describing the essential structure.

The first author and interviewer, HN, is a midwife with experience supporting women during labour and birth. The other three researchers are senior lecturers in midwifery science with long experience of clinical work at labour wards. During the analysis, as part of the bridling process, there was an ongoing discussion on whether this was the best way to understand the phenomenon in focus or if there might be different ways of understanding it.

### Ethical approval

Ethical approval was granted by the Regional Ethics Board in Gothenburg, Sweden (DNR: 285–14, T1066-17).

## Results

The essence of the phenomenon of women’s lived experiences of induction of labour in late- and post-term pregnancy can be described as the *labour becomes another journey* instead of the intended journey. *Another journey* means that the labour process is induced, instead of the intended journey with spontaneous onset whereby the body itself would have initiate the labour; it was not the journey that the woman had originally planned. The intended journey is now removed and replaced by another journey involving the induction, which the women reflected upon as they adapt and adjust themselves to the new journey.

*Another journey* was introduced by a research project, not by the women themselves. Nevertheless, they see the advantages of another journey, as it means that the labour will finally start. Replacing, lifting, and acknowledging the positive aspects of *another journey*, and comparing it to the intended journey, made the new journey meaningful. This created a purpose for another journey, making the induction valuable to the women.

Something is lost with *another journey* in comparison to the intended journey. Whatever the women are missing out on when embarking on another journey due to the induction tends to linger in the background as they adjust to another journey: In the back of their minds they had a feeling that something about giving birth is lost, a sense of loss at not experiencing what their bodies’ own contractions would feel like and how they would handle them, the loss of a natural birth.

The essential structure can be further described by its four constituents: *planning the unplannable, being a guest at the labour ward, someone else controlling the labour*, and *overshadowed by how it turned out*.

### Planning the unplannable

The induction was described as a possibility to *plan* for something that is *unplannable*, in the sense that the women now knew the date of the birth. The unplannable is the spontaneous onset of labour. The women did not previously know when, where, or how it would start, or even whether it would start naturally. Passing the expected due date was both physically and mentally strenuous; the women had expected to have already given birth. For them, this plan gave them a feeling of relief as they finally had a planned date for the end of their pregnancy and their baby’s arrival. It offered an end to the yearning, waiting and discomfort that comes with pregnancy, as well as the feeling of curiosity about their baby:

Well, preferably, you’d want it to start automatically. However, now … I could tell she was done baking. And I wanted so much for her to come out. (Charlotte, third child)

*Planning the unplannable* by taking part in the research project presented an opportunity to have labour induction in gestational week 41, a week earlier than the routine care at the participating hospital. It was considered a benefit to meet one’s child earlier thanks to the induction. Martina (second child, induction week 41) expressed this by saying it felt “like I’d won the lottery”.

It was a relief to be given a plan for the end of one’s pregnancy and a final due date. Emma (second child), who was randomized to expectant management until gestational week 42, expressed “First I was disappointed for about ten seconds. But then *[*I thought*]*, oh thank God, how nice, now I know, now I know when I’ll be induced.” Sarah (first child) described feelings of relief, when she was randomized to induction in gestational week 41: “I got really happy while sitting on that couch. Wow, now I’ll get help. Now I’ll finally give birth.”

The women were pleased to be induced for labour in order to be able to plan the unplannable, as they could now let go of some of the stress and uncertainties they felt were associated with the spontaneous onset of labour. They described it as a way to find something positive in the situation when the spontaneous onset did not take place, and as a way to cope with the situation. The women would not be at risk of being sent home due to not being considered to be in active labour, of being referred to another hospital due to lack of space, or of not making it to the ward in time and giving birth in their car or an ambulance. Thanks to the planned induction, they were guaranteed a bed at the labour ward:

Also, I was so nervous before that it would … before I was induced, that it would happen so fast that I wouldn’t be able to get in on time [to the labour ward]. It was also something that I was thinking about during the whole pregnancy” … “It felt pretty safe to get this induction. It meant I would actually be there when it was about to happen [the labour]. (Marie, third child)

Labour induction was perceived as practical as it allowed the women to know when they would go into labour, making the planning easier as they would not have to call the babysitter or dog watcher on short notice. It offered a sense of relief to be able to plan what is usually unplannable. It caused them less stress and eliminated a source of worry that they could now let go of:

On the one hand it was very handy to know, to have a date confirmed. To have a babysitter here an hour before we left and to have breakfast on Sunday morning. On a Sunday when Granddad was off work as well. It was as good as it could be. (Martina, second child)

The women described how their surrounding context affected their decision to take part in the study and choose the induction. This could involve experiences of induction of labour among friends and family, or if the midwife suggested or opted for them to take part in the study. Karolina (first child) had originally aimed for a natural birth but chose to take part in the study because her midwife and family were concerned: “*It made me worried that others were worried”.*

### Being a guest at the labour ward

*Being a guest at the labour ward* entails being present at the labour ward, not as a woman in labour but instead just waiting for the more active part of the labour to start. The women described being at the labour ward during the induction, before the active part of the labour had started, as a long and tiresome wait. At this stage of the induction, the women tended to not see the staff other than when it was time for examinations or check-ups. They were encouraged to rest to prepare themselves for active labour. However, according to the women, in reality there was no actual rest. Their resting time was interrupted by examinations and routines at the labour ward, making them tired when the active part of labour was about to begin. Additionally, they described it as a special feeling being at the labour ward waiting for the contractions to start, aware that they were about to voluntarily go into labour. This was described as by Charlotte, who was having her third child:

At first, it was kind of weird to be induced. Before you were like … you get into the pain as it sneaks up gradually. Now you have to wait for it to come. So you get quite nervous. (Charlotte, third child)

*Being a guest at the labour ward* can be a special situation if there are no available rooms and/or if there is a shortage of staff. With spontaneous onset, women coming to the ward in active labour and would be prioritized over others due to their contractions. However, with a planned induction women did not get the same attention as women in active labour, who have a more urgent need for help. This could lead to the woman and her partner having to wait a long time for a room. Karin, pregnant with her first child, explained how she felt about this: “They kept forgetting about us”. The women described that they understood that women in active labour should be prioritized, but that this negatively affected their own wellbeing and experiences:

By then I’d been sitting in the waiting room just watching that TV and I was kind of pretty tired already. Well, it was sort of like when you’re sitting and waiting at an airport, just waiting. You get sort of bone-tired from just sitting there staring at the wall. (Patricia, first child)

*Being a guest at the labour ward* made the women reflect upon the existential life-changing event of childbirth for the individual families while it was an everyday event for those who worked there. This is described by Ariel, whose oxytocin drip was postponed so that the staff could take time off to have lunch:

We’re experiencing one of our most magical moments and right here where we are, our life is put on pause. But somehow, in some way, it was a nice contrast to that. This is actually someone’s place of work. Of course they should go off and have lunch and a break. (Ariel, second child)

At the same time as the women were trying to rest, they were also trying to contribute to getting the labour started. The women describe that they tried to activate themselves by walking in the corridors, eating, watching movies, and resting, trying to feel more at home at the labour ward, even though it was an unfamiliar environment. They describe how these early stages of the induction could have taken place at home, which would have made it easier for them to relax, rest, and move more freely. They expressed how they might have been able to relax in a more comfortable way at home:

I may have just felt more comfortable at home. Even if I’d just been lying on the sofa watching telly, you’re sort of more relaxed in some way, when you have your own belongings [around you]. And even if you were to … well it’s not as if you’d start cooking or something like that, but it’s more that you feel a bit more confined when inside the hospital. Well, you have that bed there and then there’s an armchair, that’s it. (Nina, first child)

When walking up and down in the corridors, the women were able to hear other women in active labour. This made them long for that moment and contemplate what it would be like to be the person who was giving birth:

And I could sort of hear the women screaming and so on. And then I thought, how is their pain, if they’re screaming like that? In my thoughts, I was a bit frightened, but still at ease, lucky her, now she’s given birth to her baby. I was longing for that too, for that phase. (Sarah, first child)

### Someone else controlling the labour

*Someone else controlling the labour* means that when the women accepted and consented to labour induction, they handed themselves over to the maternity personnel at the labour ward. The women described trust in the personnel to know what best practice was and how to handle labour and birth, handing over the control of the labour without question. As Karolina (first child) expressed it: “*Now we don’t have to worry anymore. Now you’re keeping an eye on me.”*

The women gave consent to the induction, but from that moment on no further consent was given and they submitted to both written and unwritten instructions and routines at the labour ward:

Then there was a standard procedure, that then we do like this, then we break the water and like that. So, they did it. *(Karolina, first child)*

The women described how the process of labour and contractions was controlled by the maternity personnel; they were the ones who determined whether the contractions were sufficiently strong and frequent. Charlotte, who was pregnant with her third child, described the moment she started having contractions:

“And then I thought it started to feel like this sort of cosy pain. But not enough, apparently, for the midwives, so they put me on the oxytocin drip.” *(Charlotte, third child)*

*Someone else controlling the labour* describes how the women put their own estimations aside in favour of the hospital’s routines for determining progress. Frida (first child) described when her water broke: “Oh, now the water’s broken. And I was surprised that they didn’t agree. They weren’t as impressed as I was. That was weird.” The midwives checked whether it was a rupture of the membranes by giving her a pad to see if the water would continue to drip. “But then they came to the conclusion that the water had broken.”

The women also expressed that the staff at the labour ward took it for granted that the women would follow their routines and recommendations. Sarah, pregnant with her first child, clearly expressed that she had refused a balloon catheter as an induction method, but was persuaded to have this intervention despite her unwillingness. This led to a very negative, traumatic experience for her:

So, then she started telling me how this catheter method would proceed. And then I told her again that my wish was to be given tablets instead. But she was like, we’ll start with this. And so she did. *(Sarah, first child)*

*Someone else controlling the labour* describes a situation in which the women experienced being part of a schedule and the maternity personnel monitoring the women’s adherence. There was no time for a pause. Frida, pregnant with her first child, wanted very much to have a bath during her labour:

Well, I tried to go and have a bath. But then they told me ‘no, you can’t’. But we can take it off [the oxytocin drip] I said, I don’t mind if there’s a pause. But they didn’t want that. So, I wasn’t allowed to have a bath. *(Frida, first child)*

The women considered it better to have a short, consistent labour than to risk having a long labour. If the labour was short, even if it was intense, the women expressed gratitude that it was not taking a long time and accepted that the staff accelerated it. The women adjusted to what was happening and simply tried to follow along. They felt safe that the staff had control over the labour, and handed themselves over to their care:

Well, a baby’s about to arrive, let’s speed it up [the oxytocin drip]. Hi ho hi ho, sort of. It wasn’t … well, it felt good and safe, sort of. But it was sort of hi ho let’s go. But it was also my way, to be open to it, to what they … to totally submit myself. *(Emma, second child)*

*Someone else controlling the labour* also refers to the medications and methods used during the induction. The women talked about the pros and cons of the induction method for their bodies, and considered it more natural to use mechanical methods for induction. If their bodies responded with contractions, they considered the method to be a good one. If there were no concrete, clear signs that labour had started, they perceived that the labour was proceeding too slowly and that nothing was happening. It could be that a chosen method was effective as the cervix softened and ripened somewhat, but they still did not consider it as effective as other methods that could give more tangible signs that labour was starting. They considered contractions or their water breaking to be more specific signs that labour had started. This meant that there was no turning back and was a definite sign to them that the induction was working. This is what they had been waiting for:

And I think it was then that the water broke, or maybe she broke the water. Anyway, there was a ton of water and then it felt like Yay! Finally, it’s happening, it’s really started. *(Karin, first child)*

The women handed themselves over to the technology, equipment, and medicine, describing that they were surrounded by a great deal of equipment that affected their mobility. The situation grew very clinical, and they described feeling more like a patient than a woman giving birth:

Then you got tangled up in that darn IV tube all the time, so you got caught in everything. And then the fetal monitor. So I felt very attached. I wasn’t myself. It was more like if you were sick. I wasn’t there because I was sick, but because I was going to give birth to a baby. But it felt more like if you were a patient, sort of. *(Karin, first child)*

### Overshadowed by how it turned out

*Overshadowed by how it turned out* is a reflection by the women on the labour and birth in relation to the induction. The women reflected on their experience and on how the induction might have affected the experience and outcome. They expressed that they would have wanted the labour to start spontaneously as they had wished for a natural birth, and said it was similar to missing out on something but not knowing just what. They had thoughts concerning how natural contractions would feel compared to those that were controlled by the maternity personnel and medications. Would there be a difference? The women were curious as to how their bodies would have responded during a natural birth and how they would have coped during a spontaneous onset:

The difference in pain when the body gets to handle it by itself [the birth], compared to when someone else is deciding how many contractions you should have. *(Nina, first child)*

The women reflected on how their body was forced and stressed into labour, and to give birth whether or not their body was ready. Their baby may have been forced out although it was not ready to come out. They also described this as their body needing help to understand that it needed to give birth:

Well, we sort of forced the whole thing to happen with a lot of medicine, it felt like. So, it got to be like … so then it [the body] protested sort of, like ‘No! I don’t want to do that’. And then, of course, it got quite tough when it was finally time to push. Because my brain was involved, so then I had to force my body even more. That’s probably why it ended with an episiotomy and everything. Because even though I was fully dilated, it wasn’t … I wasn’t ready in some way. This is how it felt. *(Karin, first baby)*

They compared the pros and cons when reflecting on their induction and labour. Their experience of the induction was overshadowed by how the labour and birth turned out, their encounters with the maternity personnel, and any complications:

Already from the beginning I was terrified about the labour. I thought the induction would help me” … “I think I thought that it would be safer that I would have staff with me the whole time. I thought it would be quicker. But it didn’t turn out like that. *(Sarah, second child)*

In addition, the women expressed disappointment and distrust regarding their bodies’ ability to initiate labour and birth. They felt that the induction had been necessary for their baby to be born:

I don’t trust my body to tell me when it’s time for the baby to come. Because it feels like my body doesn’t tell me such things. It might, but it doesn’t feel like that as I haven’t experienced it myself. Thus, I think my babies might just stay inside and wither and then die. *(Emma, second child)*

In retrospect, the women expressed that they may have also felt that it could have waited; that the labour could have started when their body and baby were ready. Likewise, they expressed how, in a future pregnancy, they might be more patient and have greater trust in their bodies:

It’s a treat to get to start earlier, to be induced. But now I’d probably be more like: If the baby isn’t due, or the body isn’t ready, then you just need to wait, sort of” … “I think I trust my body more now, whereas in the first labour I trusted healthcare more. That on my second go, I might await it a little further. *(Sanna, first child)*

The induction became part of the labour, and the experience of induction was overshadowed by how it turned out. The women described that their bodies were capable of giving birth even though their labour was controlled by the staff and medications, and even though there was no spontaneous onset. Despite everything, it all worked out in the end because their baby was born:

Well because it didn’t happen fully naturally—that it may not have been the plan [to give birth at this time]—but still everything’s working as it should and my body’s working like it’s supposed to. That’s pretty amazing. (Charlotte, third child)

## Discussion

The findings from this study show that the essence of women’s experiences of induction of labour in late- and post-term pregnancy entails *labour becoming another journey* instead of the intended journey. The women adopt and adjust to *another journey* instead of the spontaneous onset of labour, the intended journey. However, something about giving birth might be lost. Four constituents further describe the essence: *planning the unplannable, being a guest at the labour ward, someone else controlling the labour*, and *overshadowed by how it turned out*.

The constituent *planning the unplannable* describes how the women, when they knew the date and time for the induction of labour, could let go of uncertainties they associated with the spontaneous onset of birth. In a study by Wessberg et al. (2017), it is described how women experienced late-term pregnancy as a time that could be dominated by negative feelings and thoughts, difficulties associated with waiting for labour and unmet expectations. In our study, the women expressed being relieved at receiving a date for the induction of labour. They described it as physically and mentally strenuous to pass their due date. With the induction, though, they had a plan: The journey towards meeting their child had finally started, even though induction was another journey than the expected one. The individual woman’s needs during pregnancy and childbirth, supporting her autonomy and involving her in the care process, are focused on in woman-centred care (Brady et al., 2019; Eri et al., 2020). Lundgren and Berg (2007) describe how the midwife can enable the woman to participate in the childbearing process through communication, openness, a reciprocal giving of oneself, and shared responsibility. When women are given information and knowledge, they receive affirmation and become more involved in the care process in a mutual relationship. To enable woman-centred care, it is important for the midwife to have an understanding of the woman’s needs and experiences in late-term pregnancy.

The birth experience is held to have profound and significant meaning for women and their families (International Confederation of Midwives, 2014). Childbirth is not an everyday experience for the individual family, and needs to be treated with deep respect by healthcare personnel (Crowther & Hall, 2015). This is observed in the constituent *being a guest at the labour ward*: When women came to the ward for the induction, they reflected on the birth of their baby as an existential, life-changing event for them and their partner. However, it needs to be reflected upon how it affects the childbirth experience and women’s autonomy when they have to wait a long time to receive care or are forgotten, or when their intended rest is constantly interrupted by examinations and labour ward routines. *Being a guest at the labour ward* and experiencing that they did not yet have a self-evident place there as a labouring woman raises questions about how this affects women’s birth territory. Fahy and Parratt (2006) describe the birth territory as the psychological and physical features that create a woman’s individual birth space and use of power within the birth environment (Fahy & Parratt, 2006). How can women make an individual space for birthing if they experience that they are guests at the labour ward?

In their ethnographic study, Newnham et al. (2017) describe work at a labour ward focusing on risk, taking a pathogenic perspective on labour and birth. This led to women being rushed through labour and birth with a focus on monitoring, managing, and controlling risks rather than on the normal process of labour and the birthing woman’s needs (Newnham et al., 2017). In their study, Goldkuhl et al. (2021) describe that some women took on a passive disposition during labour and that the care provider’s role tended to be authoritative with a focus on guidelines and interventions, which caused the focus on the woman’s agency, needs, and experience of labour to be subordinated to those of the institution (Goldkuhl et al., 2021). The medicalization of childbirth refers to a more risk-oriented view of childbirth as a condition that can be evaluated through measurements and controlled through medical interventions, rather than a physiological process and social event. In a medicalized setting, the personnel have the authority while patients tend to relinquish their preferences and defer to those of the personnel. In such a setting, the individual’s preferences and needs are often subordinated to the standardized processes and guidelines (Davis-Floyd, 2001). This was seen in our study: The constituent *someone else controlling the labour* is related to the medicalization of childbirth. The women handed themselves over to the staff as they are the ones who “know best”, and experienced that they were not listened to when they objected to something or wanted to do something other than what was suggested, e.g., not wanting a specific induction method or wanting to have a bath. This can lead to a more negative childbirth experience, with a negative impact on the health of the woman and on her baby and family.

There is more to childbirth than what is observable and known (Crowther & Hall, 2015). In the essence *labour becomes another journey*, the women described how something might be lost with another journey. This is further noticeable in the constituent *overshadowed by how it turned out*, in the women’s wish for a natural and spontaneous onset of birth. However, it was difficult for the women to put into words what they are missing out on. Even though the labour started with an induction, the women expressed a wish to experience the normal progression of labour and curiosity about spontaneous and natural childbirth. It has been noted in previous research that most women value and hope for a natural labour and birth that enables them to use their own capacity to give birth (Downe et al., 2018). Women’s psychological experience of spontaneous and natural childbirth in a supportive and empathic environment has been described as an empowering journey (Olza et al., 2018). Providing woman-centred care has been described as a balancing act in seeing the normal in the abnormal, meaning enabling physiological and technical approaches to exist side-by-side (Berg et al., 2012). As induction per se is an intervention, this presents a challenge for maternity care providers when it comes to how we can still support the normal progress in labour and make room for the women to assume their proper place in the labour room.

### Strengths and limitations

The dominant quantitative research methods are not sufficient for fully understanding complex phenomena involving human health. Using philosophy enables us to take a step back and see the reality from a different perspective. The lifeworld theory offers an approach to how to relate to our world, how it appears, and its significance to us. The results from a qualitative study may not be generalizable. However, they can be used and transferred to other contexts by interpretation (Dahlberg et al., 2008). Hence, our findings can be more or less relevant in other contexts depending on how the induction of labour is managed and on the cultural understanding of labour and birth.

This study was undertaken with a small group of women who participated in the larger randomized SWEPIS study, in which 22% of women who were eligible during the study period participated (Wennerholm et al., 2019). The small subgroup of women interviewed in this study represent a variety of the women participating in SWEPIS when it comes to induction method, labour outcome, parity, and age. The women all expressed that their reason for participating was that they hoped for induction of labour one week earlier than routine care offered. It might be that women not participating in SWEPIS might be more neutral or negative in their attitudes towards being induced. Previous studies investigating pregnant women’s reasons for participating in randomized trials have shown that the intervention being considered favourable and not available outside the trial can be a reason for participating in a study (Monteiro et al., 2019; Oude Rengerink et al., 2015). This needs to be considered when interpreting the results of this study to other contexts.

## Conclusion

The essence of the phenomenon of women’s lived experiences of induction of labour in late- and post-term pregnancy can be understood as labour becoming another journey than the intended spontaneous onset of labour that they had hoped for. The women were relieved to finally know the date their labour would start, and adapted and adjusted to the new conditions of labour by acknowledging and highlighting what would be more positive about the journey they would end up having. However, at the same time, they felt that something might be lost when it came to giving birth.

A medicalized view of childbirth is expressed in the results. The women handed themselves over to the maternity personnel, trusting them to know best practice and allowing them to control the labour. When labour is induced, the maternity personnel face a challenge to facilitate and support women in making informed choices and decisions regarding their care, to involve them in the process, and to support their normal progress and not rush them through labour and birth.
